# KU-BdSL: An open dataset for Bengali sign language recognition

**DOI:** 10.1016/j.dib.2023.109797

**Published:** 2023-11-11

**Authors:** Abdullah Al Jaid Jim, Ibrahim Rafi, Md. Zahid Akon, Uzzal Biswas, Abdullah-Al Nahid

**Affiliations:** a Electronics and Communication Engineering Discipline, Khulna University, Khulna 9208, Bangladesh; b Computer Science and Engineering Department, University of Global Village, Barishal 8200, Bangladesh

**Keywords:** Bengali sign language, Computer vision, Deep learning, Machine learning, Sign language recognition

## Abstract

Sign language is a form of communication medium for speech and hearing disabled people. It has various forms with different troublesome patterns, which are difficult for the general mass to comprehend. Bengali sign language (BdSL) is one of the difficult sign languages due to its immense number of alphabet, words, and expression techniques. Machine translation can ease the difficulty for disabled people to communicate with generals. From the machine learning (ML) domain, computer vision can be the solution for them, and every ML solution requires a optimized model and a proper dataset. Therefore, in this research work, we have created a BdSL dataset and named `KU-BdSL', which consists of 30 classes describing 38 consonants (‘banjonborno’) of the Bengali alphabet. The dataset includes 1500 images of hand signs in total, each representing Bengali consonant(s). Thirty-nine participants (30 males and 9 females) of different ages (21–38 years) participated in the creation of this dataset. We adopted smartphones to capture the images due to the availability of their high-definition cameras. We believe that this dataset can be beneficial to the deaf and dumb (D&D) community. Identification of Bengali consonants of BdSL from images or videos is feasible using the dataset. It can also be employed for a human-machine interface for disabled people. In the future, we will work on the vowels and word level of BdSL.

Specifications Table

**Table:** 

Subject	Data Science
Specific subject area	Bengali sign language (BdSL) dataset for automatic recognition of Bengali consonants.
Data format	Raw Annotated.
Type of data	Image files.
Data collection	Multi-scaled and sized images are captured using multiple smartphones.
Data source location	• Institution: Electronics and Communication Engineering Discipline, Khulna University • City/Town/Region: Khulna-9208 • Country: Bangladesh
Data accessibility	Repository name: KU-BdSL: Khulna University Bengali Sign Language dataset Data identification number: 10.17632/scpvm2nbkm.4 Direct URL to data: https://data.mendeley.com/datasets/scpvm2nbkm/4

## 1 Value of the Data

• The dataset is based on static single hand gestures that are easy to adopt, which is a medium of communication for the D&D society.
• This dataset can be utilized for a cost-effective, interactive, and easy to use Bengali language translator from sign language. Besides it can satisfy the need of special education system for D&D society.
• The dataset has proper ethics approval and consent from the participants (all of them are adults who provided consent), which makes it more accessible for the research community.
• The dataset can be used for validating a model trained with an existing dataset, and vice versa. Also, researchers can mix this dataset with another dataset to improve the adaptability of a model to new data.
• Mixing this dataset with the existing dataset will increase the sample size, more importantly, it will vary participants, lighting conditions, and image capturing techniques, and will remove biases associated with the dataset.
• ML models are data-dependEnt, hence newer datasets will help improve their accuracy, performance, robustness, etc.

## 2 Background

The existing solution for understanding the speech and hearing-impaired people mostly relies on learning sign language or seeking help from an expert. The primary obstacles to learn sign language is the willingness and efforts of people. Moreover, it takes a lot of patience and practice. Computer vision and deep learning have gone a long way in recent years. Hence, the dilemma of learning sign language is addressed by adopting deep learning. A proper dataset and an accurate deep-learning model can become the desired alternative. A large population in Bangladesh is suffering the curse of hearing impairment (around 2.4 million BdSL users in Bangladesh [1]). Therefore, we have worked on this dataset to contribute towards the betterment of the D&D people using BdSL. The sole purpose of this study is to make a proper dataset of Bengali consonants (‘banjonborno’) so that it can aid an ML model. Labelled BdSL datasets are scarce, so we wanted to make one. Sometimes, all we have is a preprocessed dataset, which limits the usability of a dataset. Hence, we wanted to provide the raw dataset so that the researcher may modify it. Other sign languages are more developed than BdSL, which is another motivation for the creation of this dataset.

## 3 Data Description

There are numerous ways to express sign languages, particularly for the D&D community in Bengali sign language (BdSL). The BdSL is categorized into two major groups depending on whether the hand is moving or stationary- static sign language [2,3] and dynamic sign language [4,5]. Based on the number of hands employed, these two categories can be further subdivided into two sub-categories. By combining these expressing techniques of BdSL, we get four approaches (e.g., static single handed and dynamic double handed method) using which we can represent the same sign. Also, there are 38 consonants and 11 vowels in Bengali alphabet, which is greater than the most languages such as English (26), French (26) [6], Greek (24) [7], Arabic (28) [8], Russian (33) [9]. Moreover, Bengali has an enriched grammar and large vocabulary [10], which makes it one of the difficult languages to express using hand signs. Researchers are interested in the single-hand strategy because the double-hand approach is antiquated and difficult. Therefore, single-hand BdSL strategy is prioritized for our dataset creation.

We have represented 38 consonants (‘banjonborno’) from Bengali alphabet using the static single-hand approach and created a dataset termed ‘KU-BdSL’. The Bengali consonants can be represented by 30 hand signs [11]. Thus, in this dataset, the consonants are divided into 30 classes, each with 50 images, for a total of 1500 images. The overall number of consonants exceeds the number of classes because of few hand gestures in BdSL representing multiple letters.. We have maintained a constant number of samples in each class so that the dataset does not become imbalance. Imbalance dataset causes dilemmas like biased models, poor generalization, classification threshold selection, etc. [12]. We have selected a fixed number of samples in each class so that the users of the dataset require no additional efforts to make this a balanced dataset. Methods such as K-means clustering [13] can be helpful for recognition of this type of data.

There are 39 participants (30 male, 9 female) in the creation of the dataset, aged between 21 and 38 years. The participants are from eight different divisions of Bangladesh. The age range of the female participants is 21–25 years, and for the male participants, it is 21–38. The average age of the male and female participants is 23 years and 23.97 years, respectively. All the participants of this research work are from Bangladesh and Bengali speaking people. Most of them are familiar with sign language but not regular users of BdSL. Therefore, we have shown them BdSL hand sign references from [11] and [14]. They quickly learned and imitated the hand gestures. Those who agreed to work voluntarily became the participants of this research, no financial or social benefits were provided to them.

We have used two smartphone cameras, one has 48 megapixels (LE2101), and the other one (iPhone 7 Plus) has 12 megapixels. First, the participants have been asked to provide consent. Then, they are introduced to the BdSL hand gestures and asked to imitate them. Participants have placed their hands before a background, and the photos are taken. The background of the images is varied to remove any bias while training a model. Different images have different lighting conditions so that the model accuracy remains unaffected by the colour of an image.

First of all, the KU-BdSL folder contains three folders - AMSLD (Annotated Multi-scale Sign Language Dataset), MSLD (Multi-scale Sign Language Dataset), and USLD (Uni-scale Sign Language Dataset). These three folders hold the three variants of our data, including the raw dataset. MSLD folder contains the original dataset, which has various scale images. The images are scaled in 512×512 pixels and stored in USLD folder. A few machine learning models, such as CNN, are afflicted with input dimension difficulty, which is taking input of a predefined size. In such circumstances, this USLD can be useful. The folder names in MSLD and USLD are represented by the respective Bengali letters' Unicode value (e.g., ‘’ has a folder name of 2453, and ‘ ’ has 2433), as shown in Table 1. Each folder contains 50 images portraying the hand sign of that alphabet.

**Table 1:** Hand postures and their corresponding Bengali alphabet and folder names of KU-BdSL.

Annotating images is a time-consuming and laborious task that motivates us to create AMSLD folder. The ASMLD folder includes a folder named ‘DarknetAnnoted’ and a file named ‘obj.names’. YOLO DarkNet annotation has been taken into consideration for annotation purposes. The DarknetAnnoted folder provides images in jpeg format as well as the annotation file as a text file. With an exception in the file format, the annotation file has the same name as the image. The text files include five numerical values that correspond to the class ID, normalized x-axis value, normalized y-axis value, width, and height. The definition of these five values, necessitating for annotation, is further stated in the ‘obj.names’ file.

## 4 Experimental Design, Materials and Methods

Single-hand static pictures have formed this dataset. High-quality smartphone cameras are utilized to capture the images. The backgrounds of the images are both solid colours and multicolour to boost the robustness of the dataset. The images include only the hands symbolizing the signs, and the other objects kept abstained. The images are taken at separate daytime to vary the colour, contrast, and brightness in real-time, without using data augmentation techniques. This method simulates a real-time environment at the expense of distortion or sacrificing visual quality. The information regarding smartphones and their cameras used in our work has been presented in Table 2. We have used two smartphones (operating system: android and iOS) so that if any smartphone application [15] is developed, this dataset can be utilized.

**Table 2:** Specification of our smartphones and their cameras.

Serial Number	Brand Name	Model Name	No of Images
1	OnePlus	LE2101	289
2	Apple	iPhone 7 Plus	1211

**Fig. 1:**
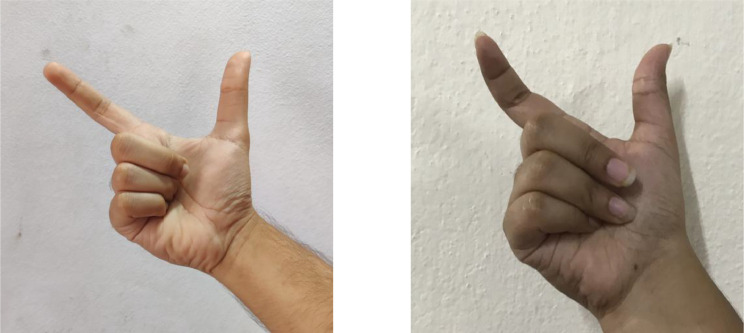
Different hand gestures for ‘Chandra bindu’.

Together with thirty males and nine females, totalling 39 people have participated in the creation of this dataset. Sometimes the participants have used one hand to imitate the hand postures and another one to capture the images. Due to the intricacy of the hand gestures, another person is necessary to capture most the images. In general, participants were briefed and taught BdSL hand signs and their meanings and were asked to copy them. When they successfully copied a hand sign, images were captured. Multiple samples have been collected, varying angles and postures from the same participant simulating real-time situations. Images have been captured under various lighting conditions. Some samples were taken under indoor conditions and for some samples, natural light was utilized. Lighting condition was varied intentionally to make the dataset more robust and give it the ability to work under various lighting scheme. While working indoors, lights are generally focused on the participants, or diffused lights were introduced. We have not taken any images against the light source. Some signs from the BdSL can vary regarding the hand gesture, which may seem identical at first glance. In those conditions, several hand postures have been adjusted, and images have been captured. For instance, both the images in Fig. 1 portray ‘Chandra bindu’ (‘ ’) from BdSL, whereas they look different in the human eye and machines. This form of the quandary has been solved by accumulating all the hand postures in our dataset.

Prior to asking for their consent, we have given each participant a form with proper instructions. Aside from the background of the research, description, participant responsibilities, absence of harms, absence of benefits, alternatives to participation (i.e., not participating), protecting your data samples, participation and withdrawal, new findings and information are also included in the consent form. We have also spoken to the volunteers and briefly explained the study before capturing the image. We have told them that this data will be publicly available for the research community towards the betterment of the D&D society. After signing the form, a participant has been instructed to imitate a hand sign (as required). Finally, only the participant's hand signs have been captured on camera. Privacy concern is one of the potential challenges, hence the meta data of the images are removed so that participant's identity remains anonymous. Also, children and senior citizens are not included due to lack of the ability of provide significant consent.

After recording all the images, we have classified 30 hand gestures from BdSL that has represented 38 consonants from Bengali alphabet into 30 classes in our dataset. Each class has 50 images, and no data augmentation methods have been applied to the original dataset. Then three variants of the dataset were made, namely- (i) Uni-scale Sign Language Dataset (USLD), (ii) Multi-scale Sign Language Dataset (MSLD), and (iii) Annotated Multi-scale Sign Language Dataset (AMSLD). The raw dataset is named MSLD.

To annotate the images, we have used a GitHub repository named Make Sense [16]. It is an open-source application licensed under the GPL-3.0 License. According to the author of [16], Piotr Skalski, to function the application locally, npm 6.x.x and node.js v12.x.x versions are required. After implementing the Make Sense locally, the images have been annotated in DarkNet annotation format manually. We have drawn the bounding boxes around the hand signs. We have also labelled the boxes after sketching them in an image. Drawing bounding boxes and labelling have given us the required x and y coordinate values of the starting point of the bounding box, height, width, and the label of the gesture. By labelling 1500 images, we have created the AMSLD variant of the dataset.

**Fig. 2:**
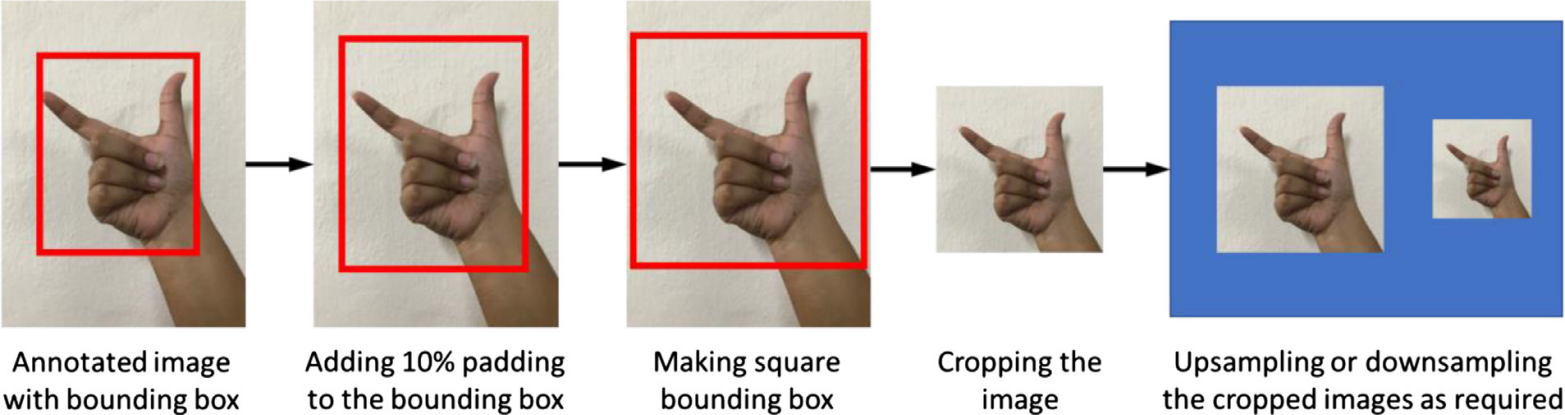
Creation of USLD variant.

The images from the AMSLD variant have been used to create the final variant, the USLD. We have obtained the height and width of the significant portion of images, the hand gestures, from the annotated files. Based on the signs' posture, the bounding boxes have been rectangles of various sizes. First, we have used 10 % padding to the existing bounding boxes, which has allowed us to incorporate more information into the USLD variant images. Then the rectangular bounding boxes have been converted into square-shaped ones. To accomplish the task, we have determined which has a lower value between the height and width of the rectangular bounding box and changed it to the other one. For instance, if the width is less than the height, the width is increased to match each other and vice versa. After completing this task, we have cropped the images based on the square bounding box information and the coordinate values to extract the desired image. Finally, we have acquired the desired image size by upsampling or downsampling the cropped image as necessary. The overall process of obtaining the USLD variant is illustrated in Fig. 2.

Ensuring the privacy of the participant's personal information is prioritized in this research. For this, we have cleared the metadata of the images, and the identities of the participants have been kept hidden. However, we have obtained the consent of the participants to use the data for research purposes by any researcher. Anything towards the betterment of the D&D society with this dataset (without financial profit) is allowed. The dataset and its variations (USLD and AMSLD) are publicly available for use, modification, and research. In the future, a proper dataset of the Bengali alphabet with numerals is intended to be created. Word and sentence level BdSL sign language dataset and its recognition can be another aspect of research.

## Limitations

Not applicable.

## Ethics Statement

The data only contains hand gestures that represent Bengali sign language and does not include personal information of the participant. It is an open access dataset with participants’ consent, and the volunteers participated willingly. Also, this study has received ethical approval from the Khulna University Human Research Ethical Clearance Committee (approval number KUECC-2022/11/40).

## CRediT authorship contribution statement

**Abdullah Al Jaid Jim:** Conceptualization, Methodology, Software, Formal analysis, Investigation, Data curation, Writing – original draft, Visualization. **Ibrahim Rafi:** Conceptualization, Methodology, Software, Formal analysis, Investigation, Data curation, Writing – original draft, Visualization. **Md. Zahid Akon:** Methodology, Formal analysis, Data curation, Visualization. **Uzzal Biswas:** Conceptualization, Methodology, Validation, Formal analysis, Writing – review & editing, Visualization, Supervision. **Abdullah-Al Nahid:** Conceptualization, Methodology, Validation, Formal analysis, Writing – review & editing, Visualization, Supervision.

## Data Availability

KU-BdSL: An open dataset for Bengali sign language recognition (Original data) (Mendeley Data).
